# Survey of Maize Rhizosphere Microbiome Using Shotgun Metagenomics

**DOI:** 10.1128/MRA.01309-20

**Published:** 2021-01-07

**Authors:** Olubukola Oluranti Babalola, Oluwadara Pelumi Omotayo, Nicholas Ozede Igiehon

**Affiliations:** a Food Security and Safety Niche, Faculty of Natural and Agricultural Science, North-West University, Mmabatho, South Africa; Georgia Institute of Technology

## Abstract

Several processes which occur in the rhizosphere make it a vital region in plant development. However, studies that examine rhizosphere microbiomes and their functional potentials remain scarce. Shotgun metagenomics was employed here to evaluate the functional potentials of the maize rhizosphere microbiome of farms in two South African provinces.

## Abstract (precis)

Several processes which occur in the rhizosphere make it a vital region in plant development. However, studies that examine the rhizosphere microbiome and their functional potentials remain scarce. Shotgun metagenomics was employed here to evaluate the functional potentials of the maize rhizosphere microbiome of farms in two South African provinces.

## ANNOUNCEMENT

Interactions between a plant and the rhizosphere microbiome are essential for enhancing crop productivity (1, 2). The study of the plant-microbe relationship in this region can improve sustainable agriculture and also aid the development of biofertilizers and biopesticides (1). Nevertheless, few studies exist that examine the maize rhizosphere microbiome and its functional capabilities; therefore, using shotgun metagenomics, this study aimed to unveil the microbial community structure and functional potentials lurking in the maize rhizosphere.

Soil samples were collected in triplicates from maize rhizosphere from two maize farms located at Lichtenburg (Fs) (25°59′40.8″S, 26°31′46.6″E) and Randfontein (Rs) (26°11′51.3″S, 27°33′18.6″′E), in North West and Gauteng provinces of South Africa, respectively, during the summer season. In order to collect the samples, maize plants were carefully excavated, and the soil loosely attached to the root was removed, while soils that were tightly attached to the roots were collected. Samples were transported on ice to the laboratory and stored at −80°C for downstream applications. From 5 g of each soil sample, DNA was extracted using the Qiagen DNeasy PowerSoil isolation kit following the manufacturer’s protocol. The extracted DNA was put through shotgun metagenomic sequencing at the Molecular Research Laboratory in Shallowater, TX. The Qubit double-stranded DNA (dsDNA) high-sensitivity (HS) assay kit (Life Technologies) was used to evaluate the concentration of DNA used for analysis prior to library preparation. The Nextera DNA Flex library preparation kit was employed for preparing the DNA libraries with the aid of the manufacturer’s user guide. A total concentration of 50 ng was used for preparing the libraries. The samples were subjected to simultaneous fragmentation and addition of adapter sequences, which were used during a limited-cycle PCR when unique indices were added. Measurement of the final library concentration was done using the Qubit dsDNA HS assay kit. The average library size was determined using the 2100 bioanalyzer (Agilent Technologies, Santa Clara, CA, USA). The libraries were pooled, diluted (to 0.6 nM), and sequenced paired end for 300 cycles using the NovaSeq system (Illumina).

Downstream analysis of the reads (totals of 18,942,494 for Fs and 15,666,716 for Rs) was carried out using the default settings of the Metagenomic Rapid Annotations using Subsystems Technology (MG-RAST) online server version 4.0.3 (https://www.mg-rast.org) (3). Quality control, which entails trimming of low-quality reads and dereplication, was performed on MG-RAST. Following this step, taxonomic classification and gene annotation were performed using BLAST-like alignment tool (BLAT) (4, 5) against the M5NR database as well as the SEED subsystem (6), which provides nonredundant integration of many databases. BLAT was used for searching protein databases using a translated nucleotide with an E value of 10^−5^, a percentage identity of 60%, and a maximum alignment length of 15 bp (default parameters were used for all software). For sample Fs, the mean read values showed 98.84% bacteria, 0.43% archaea, 0.65% eukaryotes, 0.02% viruses, and about 0.06% unclassified. For sample Rs, the mean read values showed 98.72% bacteria, 0.49% archaea, 0.70% eukaryotes, 0.02% viruses, and about 0.07% unclassified. The predominant bacterial phyla were *Actinobacteria* (23.99% to 34.38%), *Proteobacteria* (47.19% to 52.93%), *Bacteroidetes* (4.83% to 5.06%), *Chloroflexi* (2.19% to 2.93%), *Firmicutes* (2.70% to 2.76%), *Planctomycetes* (2.09% to 3.48%), and *Cyanobacteria* (1.02% to 1.50%). *Euryarchaeota*, *Thaumarchaeota*, *Chlorophyta*, and *Apicomplexa* were the most prevalent archaea and eukaryotes, and they constitute <1% of the total microbial composition. Functional classifications revealed genes involved in carbon fixation, nitrogen cycling, and phosphorus utilization in the metagenomes.

### Data availability.

The sequences were deposited and are available in an NCBI SRA data set under the BioProject numbers PRJNA678475 and PRJNA678469. Accession numbers are listed in Table 1.

**TABLE 1:** Sequence information for the analyzed soil samples

Sample^*a*^	NCBI BioProject accession no.	SRA accession no.	No. of raw sequence reads	No. of reads that passed quality control	No. of proteins predicted
Rs	PRJNA678475	SRX9510110, SRX9510109, SRX9510108	15,666,716	14,404,078	12,856,164
Fs	PRJNA678469	SRX9510019, SRX9510018, SRX9510017	18,942,494	17,309,422	15,115,042

a Fs, samples from Lichtenberg; Rs, samples from Randfontein.
